# Comparative evaluation of transverse width indices for diagnosing maxillary transverse deficiency

**DOI:** 10.1186/s12903-024-04580-4

**Published:** 2024-07-17

**Authors:** Guanchen Ye, Qi Li, Zhuoqi Guo, Xiaowen Yu, Yuchen Xu, Wanghui Ding, Huiming Wang, Mengfei Yu

**Affiliations:** Stomatology Hospital, School of Stomatology, Zhejiang Provincial Clinical Research Center for Oral Diseases, Key Laboratory of Oral Biomedical Research of Zhejiang Province, Zhejiang University School of Medicine, 166 Qiutao Road, Shangcheng District, Hangzhou, Zhejiang 310006 China

**Keywords:** Maxillary transverse deficiency, Diagnostic accuracy, Cone-beam computed tomography, ROC curve, AUC

## Abstract

**Objectives:**

This study aimed to compare and evaluate different transverse width indices for diagnosing maxillary transverse deficiency (MTD), a common malocclusion characterized by uncoordinated dental arches, crossbites, and tooth crowding.

**Materials and methods:**

Sixty patients aged 7–12 years were included in the study, with 20 patients diagnosed with MTD and 40 normal controls. Transverse width indices, including maxillary width at the buccal alveolar crest and lingual midroot level, as well as at the jugal process width, were measured. Differences between these indices and their corresponding mandibular indices were used as standardized transverse width indices. The reference range of these indices was determined and evaluated. Receiver operating characteristic (ROC) analysis was performed to evaluate their diagnostic ability.

**Results:**

The transverse width indices and standardized transverse width indices of the MTD group were significantly smaller than those of the control group, except for the jugal process width. The evaluation of the reference range and ROC analysis revealed that the difference of the maxillomandibular width at buccal alveolar crest was the most accurate diagnostic method.

**Conclusions:**

The jugal point analysis method may not be suitable for diagnosing MTD. Instead, measuring the difference in maxillomandibular width at the buccal alveolar crest proves to be a more reliable and accurate diagnostic method for MTD.

**Supplementary Information:**

The online version contains supplementary material available at 10.1186/s12903-024-04580-4.

## Introduction

Craniofacial development normally begins with the transverse dimension, followed by the vertical and sagittal dimensions [1]. Maxillary transverse deficiency (MTD) is a common malocclusion with skeletal deficiency of the maxilla, often accompanied by posterior crossbite, dental crowding, obstructive sleep apnea and other clinical symptoms, which could impacts patient’s craniofacial development and function, facial aesthetics, and quality of life [2–4]. The prevalence of MTD in children is reported to be between 13% and 23% [5], while it can reach 30% in adults [6]. The etiology of MTD is multifactorial, including congenital, developmental, traumatic, and iatrogenic factors [7]. A recent study suggests that nasal septum deviation may also a factor associated with maxillary transverse deficiency via affecting palatal vault morphology [8]. The most commonly used treatment for MTD is rapid palatal expansion (RPE) [9]. RPE applies external mechanical force to expand the midpalatal suture, leading to new bone formation and maxillary transverse width increase [10].

In the diagnosis of MTD, plaster casts are the most commonly used tools. As early as the beginning of the last century, Pont proposed measuring the sum of incisor widths (SI) to predict the ideal width of the maxillary dental arch [11]. Based on the Pont index, Schwarz et al. [12] incorporated the patient’s facial type into the evaluation (narrow, average, and wide facial types). McNamara et al. [13] suggested that the normal range of the transpalatal width is between 36 and 39 mm, and if the width is less than 31 mm, palatal expansion is needed. Since MTD often manifests as crowding of the maxillary dental arch, directly measuring transverse width of the maxilla seems natural. However, researchers gradually emphasized on the relationship between maxilla and mandible. Batwa et al. [14] suggested an optimal transverse maxillary-mandibular ratio of 1:1.1 and ratio below 1:0.9 would indicate the existence of MTD. Andrews [15] et al. proposed that if the transverse width difference between the upper and lower jaws is less than 5 mm, the patient could be diagnosed with MTD.

In MTD diagnosis, radiographs are frequently utilized as a valuable tool. Ricketts [16] introduced jugal point analysis method using anteroposterior cephalogram. The jugal point analysis measures the transverse difference between the bilateral jugal processes and the antegonial notches, which is regarded as a common assessment of skeletal transverse discrepancy. Hesby et al. [17] suggested that using jugal point analysis method is more accurate as intermolar width could be influenced by the inclination of molars.

Recently, Cone-beam computed tomography (CBCT) has been gradually applied in the analysis of dentofacial transverse widths due to its high resolution, precision, low radiation dose, and the ability to visualize and measure craniofacial structures in three dimensions [18–21]. Koo et al. [22] described basal bone widths as the distances between the bilateral centers of the root furcation points of the first permanent molars. They created the Yonsei transverse index through the measurement and analysis of CBCT data from subjects with normal occlusion. Miner et al. [23, 24] in Boston University proposed and evaluated the cone-beam transverse method, which measures the difference of maxillomandibular width at the lingual midroot level. Hwang et al. [25] measured maxillary and mandibular transverse width at buccal alveolar crest or lingual midroot level, and jugal process and antegonial notch width using CBCT images. Recently, there has also been increasing interest in comparing different transverse analysis methods. Kong et al. [26] evaluated responsiveness of three transverse analyses in CBCT during both tooth-supported and mini-screw-assisted rapid maxillary expansion. Zhang et al. [27] compared three MTD diagnosing method by assessing the intraexaminer and interexaminer reliability.

Despite various proposed methods for assessing the transverse dimension, there is no gold standard for diagnosing MTD, and there is a lack of comparative research on the diagnostic efficacy of different indices. Given that in children and young adolescents is typically considered as the optimal timing for RPE [28, 29], we collected participants from 7 to 12 years old to conduct this study. The maxillary transverse width at buccal alveolar crest [25], maxillary transverse width at lingual midroot level [23, 24], and jugal process width [16], were used as transverse width indices. The differences between these indices and their corresponding mandibular indices were used as standardized width indices.

This study aims to address the gap in existing literature by evaluating the most accurate and reliable measurement methods for diagnosing MTD. By calculating and determining the normal range of transverse dimensions in children, we seek to provide new insights and practical guidance for the diagnosis and treatment of MTD. Our research aims to enhance clinical outcomes by identifying the most effective diagnostic criteria, ultimately improving patient care and management.

## Materials and methods

### Study population

This study retrospectively collected MTD patients from the Stomatology Hospital, Zhejiang University School of Medicine. The control group was matched by gender and age. The period of recruitment was from 2016 to 2023. The research was carried out in accordance with the Declaration of Helsinki and received approval from the Ethics Committee of Stomatology Hospital Affiliated to Zhejiang University School of Medicine (No. 2022-082). Written informed consent was obtained from a parent from all participating patients. This study complied with the Strengthening the Reporting of Observational Studies in Epidemiology (STROBE) checklist. This study included participants who met the following criteria: [1] Age between 7 and 12 years; [2] Erupted maxillary and mandibular first molars, with either mixed or permanent dentition; [3] Bilateral Angle Class I molar relationships, with the mesiobuccal cusp of the upper first molar occluding within 1 mm of the buccal groove of the mandibular first molar; [4] Patients in the MTD group exhibited a lingual crossbite between the maxillary and mandibular permanent first molars in centric relation, while the control group demonstrated normal occlusion. Participants were excluded from the study if they met any of the following criteria: [1] Presence of missing teeth or supernumerary teeth; [2] Presence of cleft palate, median cyst in the palate, or other significant abnormalities; [3] History of orthodontic or craniofacial surgery; [4] Overbite or overjet exceeding 4 mm, to ensure that the skeletal transverse relationship was not skewed by vertical or sagittal skeletal discrepancies.

### Measurement of transverse indices

The CBCT scans were obtained from NewTom VGi (Italy). Skeletal and dental measurements were performed on the coronal section or reconstructed image of the CBCT scans with Dolphin Imaging software (Chatsworth, Calif). The reference planes of this study were as follows: the horizontal reference plane was Frankfort plane, which was the plane passing through the lowest point of bilateral orbit and the upper edge of the right external auditory canal. The sagittal plane was perpendicular to the horizontal plane and bisected the bilateral orbit points. The coronal plane was perpendicular to both the horizontal plane and the sagittal plane.

**Fig. 1:**
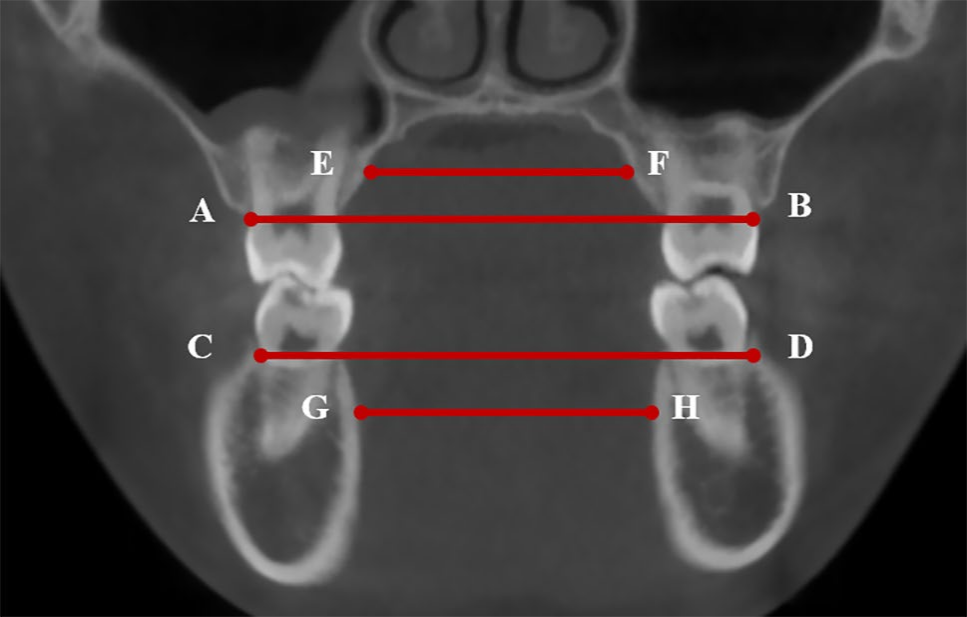
Transverse measurement in the coronal plane: **A**-**B** represents the maxillary width at buccal alveolar crest. **C**-**D** represents the mandibular width at buccal alveolar crest. **E**-**F** represents the maxillary width at lingual midroot level, with its extension line passing through the midpoint of buccal alveolar crest and the buccal root apex of the maxillary first molar. G-H represents the mandibular width at lingual midroot level, with its extension line passing through the midpoint of buccal alveolar crest and the apex of the mandibular first molar

Depending on whether the measurement object was in the maxilla or mandible, the coronal plane was adjusted to pass through the buccal groove of the right first molar in the maxilla or the buccal groove of the right first molar in the mandible [25]. The following transverse width were measured on the coronal plane (Fig. 1): [1] the maxillary width at buccal alveolar crest; [2] the mandibular width at buccal alveolar crest; [3] the maxillary width at lingual midroot level; [4] the mandibular width at lingual midroot level.

**Fig. 2:**
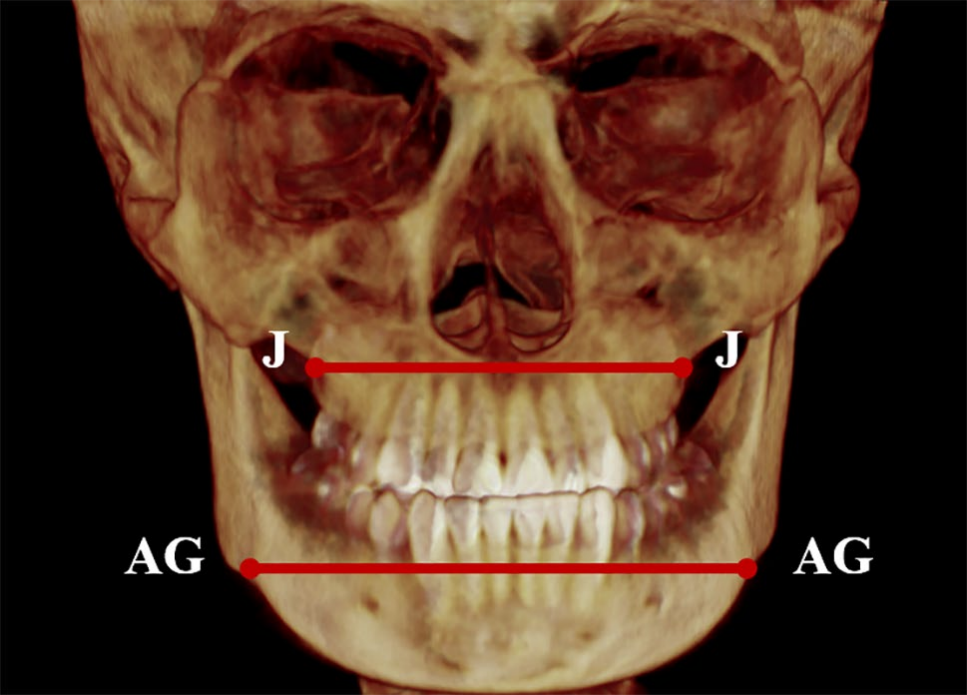
Jugal process width and the antegonial notch width in the coronal view of reconstructed 3D image: J-J width represents jugal process width; AG-AG width represents antegonial notch width

The jugal process and the anterior notch point were located on the frontal view of the reconstructed 3D images of CBCT. Jugal process width and the antegonial notch width were measured (Fig. 2).

Studies have shown that mandibular width is relatively stable and can be used as a reference [15]. In this study, the difference of transverse indices between the maxilla and mandible were used as standardized indices. The following standardized width indices were used in this study: (1) the difference of the maxillomandibular width at buccal alveolar crest; (2) the difference of the maxillomandibular width at lingual midroot level; (3) the difference of the jugal process width and antegonial notch width.

### Statistical analysis

For this retrospective study, the sample size was determined using G*Power software v. 3.1 (University of Dusseldorf, Dusseldorf, Germany) using independent t-test at an alpha level of 0.05 and a statistical power of 80% [30]. Based on a preliminary study, a minimum sample size of 10 subjects in MTD group and 20 subjects in control group was determined at an effect size of 1.16 to detect difference of the maxillomandibular width at buccal alveolar crest between the MTD group and control group. The control group was designed to be twice the size of the MTD group to enhance statistical power, ensure a stable representation of the healthy population, and achieve more precise estimates of normal range.

All data were analyzed using IBM SPSS Statistics software (IBM, ARMONK, NY, USA). Continuous variables were presented as mean ± standard deviation, and categorical variables were presented as percentages (%). The normality of data distribution was assessed using the Kolmogorov-Smirnov test, and the equality of variances between two samples was tested using the F-test. Subsequently, differences between the MTD group and the control group were analyzed using either independent samples t-test (for normally distributed data with equal variances) or Mann-Whitney U test (for data not following a normal distribution or not meeting the assumption of equal variances).

The reference range for each transverse width index was determined by calculating $\:\overline{x}$ ± 1.96 SD using data from the control group. Then, both the MTD group and the control group were included in the test set for evaluation. If the transverse width index fell below the reference range, it was diagnosed as MTD; otherwise, it was diagnosed as normal. The diagnosis results were compared to the actual grouping, with the following outcomes defined: a true positive if the actual grouping and the diagnosis results were both MTD, a false positive if the actual grouping was the control group but the diagnosis result was MTD, a true negative if the actual grouping was the control group and the diagnosis result was normal, and a false negative if the actual grouping was MTD but the diagnosis result was normal.

Positive predictive value (PPV), negative predictive value (NPV), sensitivity, specificity, and accuracy were calculated. True positive (TP), true negative (TN), false positive (FP), and false negative (FN) were used in these calculations. PPV determines the proportion of correctly diagnosed MTD cases out of all cases diagnosed as MTD, calculated as PPV = TP / (TP + FP). NPV represents the proportion of correctly diagnosed normal cases out of all cases diagnosed as normal, calculated as NPV = TN / (TN + FN). Sensitivity measures the proportion of correctly diagnosed MTD cases out of the total number of MTD cases, calculated as sensitivity = (TP) / (TP + FN) × 100%. Specificity measures the proportion of correctly diagnosed normal cases out of the total number of normal cases, calculated as specificity = (TN) / (TN + FP) × 100%. Accuracy assesses the proportion of all correctly diagnosed cases out of the total number of cases, calculated as accuracy = (TP + TN) / (TP + FP + TN + FN) × 100%.

Receiver operating characteristic (ROC) curves were plotted using MedCalc software, determining the cut point that yielded the highest sensitivity and specificity (Youden’s index), as well as calculating the area under the ROC curve (AUC), which is often referred to as the ROC score. A higher value of Youden’s Index (closer to 1) indicates a better overall performance of the diagnostic model since it reflects a more balanced trade-off between sensitivity and specificity. Likewise, the AUC represents the diagnostic performance of prediction models, where a higher AUC score signifies superior diagnostic ability.

All statistical tests were two-tailed, and *p* < 0.05 was considered statistically significant.

## Results

A total of 60 subjects were included in this study, including 20 patients in the MTD group (9 males and 11 females, with a mean age of 9.85 ± 1.73 years) and 40 individuals in the control group (18 males and 22 females, with a mean age of 9.90 ± 1.72 years) (Table 1).

**Table 1:** Demographic characteristics of participants included in the study

	MTD group (*n* = 20)	Control group (*n* = 40)	*p* value
Gender			> 0.05
Male	9 (45%)	18 (45%)	
Female	11 (55%)	22 (55%)	
Age	9.85 ± 1.73 years	9.90 ± 1.72 years	> 0.05

**Fig. 3:**
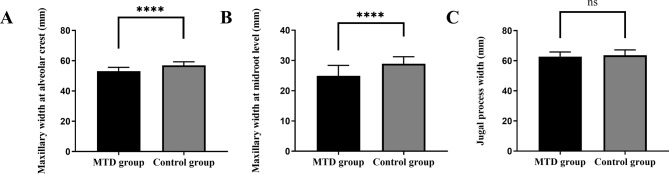
**(A)** The maxillary width at buccal alveolar crest. **(B)** The maxillary width at lingual midroot level. **(C)** The jugal process width. *Note* MTD (maxillary transverse deficiency)

Maxillary transverse width indices were compared between the MTD group and the control group, and the results showed that the maxillary width at buccal alveolar crest was significantly smaller in the MTD group compared to the control group (53.19 ± 2.47 mm vs. 56.96 ± 2.36 mm, *p* < 0.0001) (Fig. 3A); the maxillary width at lingual midroot level was significantly smaller in the MTD group compared to the control group (24.97 ± 3.41 mm vs. 28.91 ± 2.34 mm, *p* < 0.0001) (Fig. 3B); the jugal process width was smaller in the MTD group than in the control group, but there was no statistical difference between the two groups (62.80 ± 3.03 mm vs. 63.64 ± 3.58 mm, *p* > 0.05) (Fig. 3C).

**Fig. 4:**
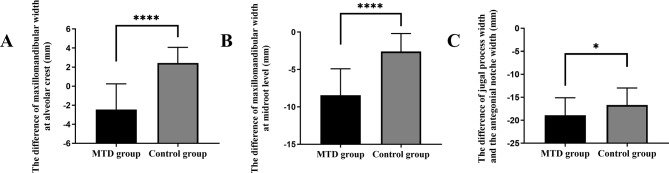
**(A)** The difference of the maxillomandibular width at buccal alveolar crest. **(B)** The difference of the maxillomandibular width at lingual midroot level. **(C)** The difference of the jugal process width and antegonial notch width. Note: MTD (maxillary transverse deficiency)

The difference of maxillomandibular width at buccal alveolar crest were significantly smaller in the MTD group compared to the control group (-2.45 ± 2.69 mm vs. 2.43 ± 1.64 mm, *p* < 0.0001) (Fig. 4A); the difference of maxillomandibular width at lingual midroot level of MTD group exhibited significantly smaller measurements compared to the control group (-8.45 ± 3.54 mm vs. -2.59 ± 2.40 mm, *p* < 0.0001) (Fig. 4B); Similarly, the difference of jugal process width and antegonial notch width in the MTD group were also smaller than the control group (-18.90 ± 3.78 mm vs. -16.69 ± 3.70 mm, *p* < 0.05) (Fig. 4C).

We also compared mandibular transverse width between the two groups. The mandibular width at the buccal alveolar crest and the antegonial notch width showed no significant difference between the MTD group and the control group (55.64 ± 2.75 mm vs. 54.53 ± 2.35 mm, *p* > 0.05; 81.69 ± 4.51 mm vs. 80.33 ± 5.11 mm, *p* > 0.05), confirming that the crossbite in the MTD group did not result from an enlarged mandibular arch but rather from maxillary hypoplasia.

Based on the data from the control group, the normal range of each transverse width index were calculated and shown in Table 2.

**Table 2:** Normal range of transverse width indices

Transverse width index	Reference range $\:(\overline{x}$±1.96 SD)
The maxillary width at buccal alveolar crest	56.96 ± 4.63 mm
The maxillary width at lingual midroot level	28.91 ± 4.60 mm
The jugal process width	63.64 ± 7.01 mm
The difference of the maxillomandibular width at buccal alveolar crest	2.43 ± 3.22 mm
The difference of the maxillomandibular width at lingual midroot level	-2.59 ± 4.70 mm
The difference of the jugal process width and antegonial notch width	-16.69 ± 7.25 mm

PPV, NPV, sensitivity, specificity, and accuracy of the predicted normal range of each transverse width index were summarized in Table 3. Accuracy, which is determined by the ratio of correctly diagnosed cases to the total number of cases, provides a comprehensive assessment of the diagnostic indices. According to accuracy, the transverse indices can be ranked as follows: the difference of the maxillomandibular width at buccal alveolar crest > the difference of the maxillomandibular width at lingual midroot level > the maxillary width at buccal alveolar crest > the maxillary width at lingual midroot level > the jugal process width > the difference of the jugal process width and antegonial notch width (Table 3).

**Table 3:** Evaluation of the reference range of transverse width indices

Transverse width index	PPV	NPV	Sensitivity	Specificity	Accuracy
The maxillary width at buccal alveolar crest	100%	76.92%	40%	100%	80%
The maxillary width at lingual midroot level	87.5%	75%	35%	97.5%	76.67%
The jugal process width	66.67%	68.42%	10%	97.5%	68.33%
The difference of the maxillomandibular width at buccal alveolar crest	93.33%	86.67%	70%	97.5%	88.33%
The difference of the maxillomandibular width at lingual midroot level	86.67%	84.44%	65%	95%	85%
The difference of the jugal process width and antegonial notch width	0%	66.10%	0%	97.5%	65%

*Note* PPV (positive predictive value); NPV (negative predictive value)

**Fig. 5:**
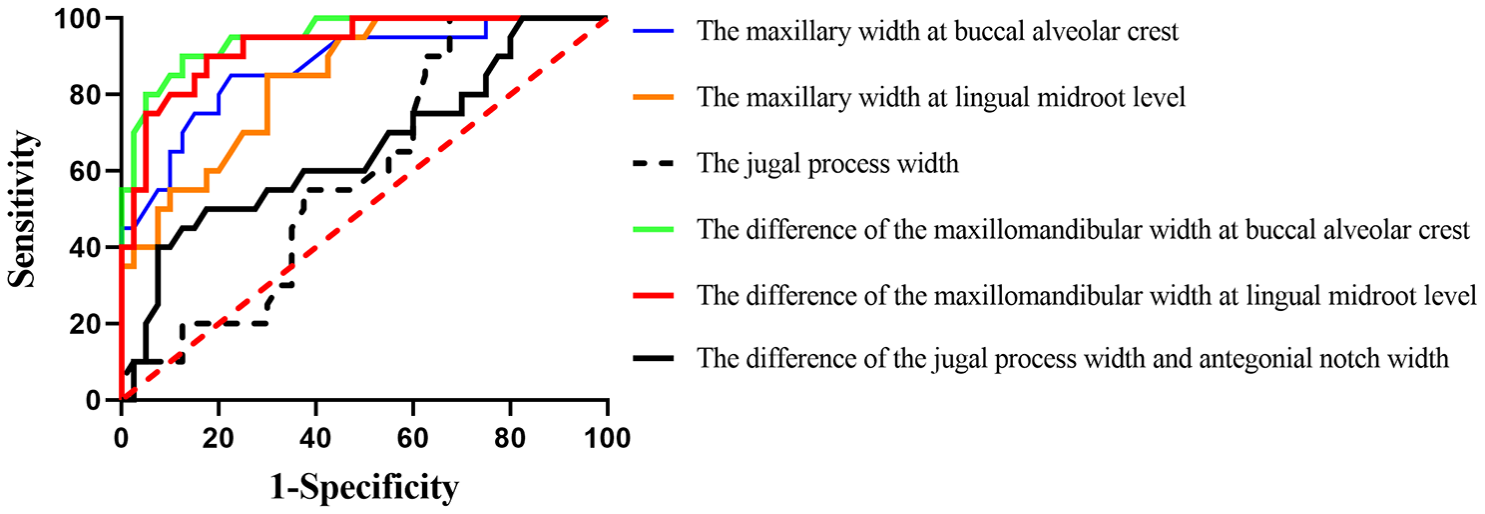
Receiver operating curves (ROCs) for the maxillary width at buccal alveolar crest, the maxillary width at lingual midroot level, the jugal process width, the difference of the maxillomandibular width at buccal alveolar crest, the difference of the maxillomandibular width at lingual midroot level, and the difference of the jugal process width and antegonial notch width

ROC curves were plotted to evaluate different transverse width indices in predicting MTD. The results showed that the Youden’s index of the maxillary width at buccal alveolar crest, the maxillary width at lingual midroot level, and the jugal process width were 0.625, 0.550, and 0.325. Additionally, the area under the ROC curve (AUC) were 0.874 (0.763–0.945), 0.841 (0.724–0.923), and 0.594 (0.460–0.710) respectively. The Youden’s index of the difference of the maxillomandibular width at buccal alveolar crest, the difference of the maxillomandibular width at lingual midroot level, and the difference of the jugal process width and antegonial notch width were 0.775, 0.725, and 0.325. The AUC were 0.951 (0.863–0.990), 0.929 (0.833–0.979), and 0.658 (0.524–0.776), respectively (Fig. 5; Table 4). In our study, both the Youden’s index and AUC indicated that the difference in maxillomandibular width at the buccal alveolar crest showed the best performance among all the transverse indices.

**Table 4:** ROC analysis of transverse width indices

Transverse width index	Youden’s index	AUC (95% CI)
The maxillary width at buccal alveolar crest	0.625	0.874 (0.763–0.945)
The maxillary width at lingual midroot level	0.550	0.841 (0.724–0.923)
The jugal process width	0.325	0.594 (0.460–0.710)
The difference of the maxillomandibular width at buccal alveolar crest	0.775	0.951 (0.863–0.990)
The difference of the maxillomandibular width at lingual midroot level	0.725	0.929 (0.833–0.979)
The difference of the jugal process width and antegonial notch width	0.325	0.658 (0.524–0.776)

*Note* AUC (Area under receiver operating characteristic curve)

## Discussion

MTD is a common malocclusion that is often accompanied by posterior crossbite and dental crowding. However, transverse discrepancies have received relatively less attention compared to sagittal and vertical discrepancies in orthodontic practice [31, 32]. Currently, the diagnosis of MTD primarily relies on assessing occlusal relationships, lacking specific diagnostic criteria based on transverse width indices. Adopting transverse indices could aid in diagnose patients with mild degrees of MTD who exhibit dental compensations without posterior crossbite and are easily missed clinically [18]. The aim of this study was to evaluate the effectiveness of various transverse width indices, determine the normal range of transverse widths in children, and contribute to a standardized framework for diagnosing MTD. We found that the difference in maxillomandibular width at the buccal alveolar crest is the most reliable indicator for diagnosing maxillary transverse deficiency (MTD).

To assess the predictive value of different analysis, we adopt the posterior crossbite as the gold standard, which could be independently verified without any other measurement or analysis. It is well recognized that if the sagittal relationship of the jaws is not within a normal range, such as a significant Class III malocclusion, the transverse relationship will be altered and might give a false impression of transverse deficiency. To address this problem, we only include clinical crossbite participants with Class I molar relationships. Patients with overbite or overjet of more than 4 mm were also excluded.

Our study determined the reference ranges of transverse indices based on the control group data and evaluated them using parameters including PPV, NPV, sensitivity, specificity, and accuracy. Among the indices, the difference of maxillomandibular width at the buccal alveolar crest demonstrated the highest NPV (86.67%), sensitivity (70%), and accuracy (88.33%), making it the most reliable method for diagnosing MTD. This index also exhibited excellent PPV (93.33%) and specificity (97.5%). The maxillary width at the buccal alveolar crest showed a high PPV (100%) and specificity (100%) but had limited sensitivity (40%), indicating that while a positive result is highly indicative of MTD, a negative result does not rule it out entirely. The maxillary width at the lingual midroot level demonstrated relatively lower sensitivity (35%) and NPV (75%), suggesting that it may not be as effective in diagnosing MTD compared to other indices. The comparison between maxillary transverse width indices and standardized width indices calibrated by mandibular width clearly highlights the importance of incorporating the mandibular width into consideration for diagnosing MTD.

The difference of jugal process width and antegonial notch width exhibited the lowest PPV (0%), NPV (66.10%), sensitivity (0%), and accuracy (65%). Jugal point analysis is a common skeletal indicator, calculating the difference between jugal point distances and antegonial notch distances. It has been suggested that this method provides a more accurate measurement of maxillary skeletal width as it is believed to be unaffected by molar inclination [17]. However, multiple studies have indicated challenges in locating jugal points and antegonial notch points on posteroanterior cephalogram, leading to significant measurement variability and making it an unreliable indicator for assessing transverse width [23, 33–36]. In a study by Legrell et al. [35], measurement deviations of antegonial notch of up to 8 mm were observed among six different observers. Precise localization of jugal points and antegonial notch points is affected by factors such as reduced clarity due to superimposition of anatomical structures in posteroanterior cephalogram and the potential impact of rotational angles during image acquisition [33, 37, 38]. Given these limitations, our study employed three-dimensional reconstructions of patients’ CBCT scans. CBCT enables precise measurements of skeletal and dental widths and positions. We established standardized reference plane for calibration and conduct the jugal point analysis on the frontal view of the reconstructed 3D images, which could minimize errors caused by inconsistent patient positioning and overlapping anatomical structures. Despite these improvements, the jugal point analysis method failed to reveal the difference between the MTD group and controls, suggesting limited sensitivity in reflecting insufficient transverse development of the maxilla and may not be suitable as diagnostic method for MTD.

This study also employed ROC curves to compare the diagnostic performance of transverse width indices. ROC curves plot sensitivity on the vertical axis and (1-specificity) on the horizontal axis based on various cutoff values. AUC of 100% indicates perfect discrimination, while 50% signifies zero discrimination, similar to flipping a coin. AUC values above 90% are considered excellent, 80–89% are good, 70–79% are fair, 60–69% are poor, and 50–59% are extremely poor [39]. The difference of the maxillomandibular width at buccal alveolar crest yielded the highest AUC (0.951) among the six evaluated indices, indicating excellent diagnostic performance.

This study’s strengths include a well-matched control group, the use of standardized transverse width indices, and the application of CBCT imaging for precise measurements. The investigation not only highlights the diagnostic efficacy of various methods but also introduces a novel approach to evaluating these indices. The study’s innovative comparative methodologies enhance the understanding of transverse deficiency diagnosis and establish a benchmark for assessing transverse indices. However, the study is limited by its small sample size and retrospective design, primarily due to the rarity of children with posterior crossbites. Future research should include larger sample sizes and milder MTD patients with buccally tipped maxillary molars, rather than just those with crossbites. This will allow for the assessment of the accuracy of indices across varying severities of MTD. Additionally, stratifying children into age groups will help establish normal range values for each age group, thus enhancing the precision of MTD diagnosis and treatment guidance.

## Conclusion

The study’s findings emphasize the clinical relevance of transverse width indices in the diagnosis of MTD and the following conclusions were drawn:

1. The jugal point analysis method, commonly used as a skeletal index, may not be suitable for diagnosing MTD.
2. Accurate diagnosis of MTD need incorporate the mandible in the measurements and calibrate the maxillary transverse width.
3. The difference of the maxillomandibular width at the buccal alveolar crest, offers a more reliable and accurate approach for diagnosing MTD. Future projects will involve larger sample sizes and test the analysis with the severity of MTD varies.

### Electronic supplementary material

Below is the link to the electronic supplementary material.

Supplementary Material 1

## Data Availability

The datasets used and/or analysed during the current study available from the corresponding author on reasonable request.

## References

[1] Cortella S, Shofer FS, Ghafari J. Transverse development of the jaws: norms for the posteroanterior cephalometric analysis. American Journal of Orthodontics and Dentofacial Orthopedics: Official Publication of the American Association of Orthodontists, its Constituent societies, and the American Board of Orthodontics. 1997;112(5):519–22. 10.1016/s0889-5406(97)70079-9 9387839

[2] Thuler E, Seay EG, Woo J, Lee J, Jafari N, Keenan BT, et al. Transverse Maxillary Deficiency predicts increased Upper Airway Collapsibility during Drug-Induced Sleep Endoscopy. Otolaryngol Head Neck Surg; 2023. 10.1002/ohn.258 36939430

[3] Brunetto DP, Moschik CE, Dominguez-Mompell R, Jaria E, Sant’Anna EF, Moon W (2022). Mini-implant assisted rapid palatal expansion (MARPE) effects on adult obstructive sleep apnea (OSA) and quality of life: a multi-center prospective controlled trial. Prog Orthodont.

[4] Bariani RCB, Bigliazzi R, Medda MG, Micieli APR, Tufik S, Fujita RR et al. Changes in behavioral and cognitive abilities after rapid maxillary expansion in children affected by persistent snoring after long-term adenotonsillectomy: a noncontrolled study. American Journal of Orthodontics and Dentofacial Orthopedics: Official Publication of the American Association of Orthodontists, its Constituent societies, and the American Board of Orthodontics. 2024;165(3):344–56. 10.1016/j.ajodo.2023.10.011 38142392

[5] Kurol J, Berglund L (1992). Longitudinal study and cost-benefit analysis of the effect of early treatment of posterior cross-bites in the primary dentition. Eur J Orthod.

[6] Proffit WR, White RP (1990). Who needs surgical-orthodontic treatment?. Int J Adult Orthodon Orthognath Surg.

[7] Betts NJ, Vanarsdall RL, Barber HD, Higgins-Barber K, Fonseca RJ (1995). Diagnosis and treatment of transverse maxillary deficiency. Int J Adult Orthodon Orthognath Surg.

[8] Jongkhum N, Arayasantiparb R, Boonpratham S, Saengfai NN, Chaweewannakorn C, Satravaha Y, et al. Association between nasal septum deviation and transverse maxillary development: a retrospective cross-sectional study. Am J Orthod Dentofac Orthopedics: Official Publication Am Association Orthodontists Its Constituent Soc Am Board Orthod. 2023;164(4):575–83. 10.1016/j.ajodo.2023.03.017 37212766

[9] Vyas RM, Jarrahy R, Sisodia M, Jourabchi N, Wasson KL, Bradley JP (2009). Bone-borne palatal distraction to correct the constricted cleft maxilla. J Craniofac Surg.

[10] Ronay V, Miner RM, Will LA, Arai K. Mandibular arch form: the relationship between dental and basal anatomy. American Journal of Orthodontics and Dentofacial Orthopedics: Official Publication of the American Association of Orthodontists, its Constituent societies, and the American Board of Orthodontics. 2008;134(3):430–8. 10.1016/j.ajodo.2006.10.040 18774089

[11] Rykman A, Smailiene D (2015). Application of Pont’s index to Lithuanian individuals: a pilot study. J Oral Maxillofacial Res.

[12] Schwarz AM, Gratzinger M. Removable Orthodontic Appliances. J Clin Orthod Jco. 1966;5(7). 5284861

[13] McNamara JA. Maxillary transverse deficiency. Am J Orthod Dentofac Orthopedics: Official Publication Am Association Orthodontists Its Constituent Soc Am Board Orthod. 2000;117(5):567–70. 10.1016/s0889-5406(00)70202-2 10799117

[14] Batwa W, Baeshen HA (2018). Use of Interarch Width ratio to measure transverse relationship: a New Method to measure and assess Interarch Discrepancy. J Contemp Dent Pract.

[15] Andrews LF. The 6-elements orthodontic philosophy: treatment goals, classification, and rules for treating. American Journal of Orthodontics and Dentofacial Orthopedics: Official Publication of the American Association of Orthodontists, its Constituent societies, and the American Board of Orthodontics. 2015;148(6):883–7. 10.1016/j.ajodo.2015.09.011 26672688

[16] Ricketts RM (1981). Perspectives in the clinical application of cephalometrics. The first fifty years. Angle Orthod.

[17] Hesby RM, Marshall SD, Dawson DV, Southard KA, Casko JS, Franciscus RG (2006). Transverse skeletal and dentoalveolar changes during growth. Am J Orthod Dentofac Orthop.

[18] Podesser B, Williams S, Bantleon HP, Imhof H (2004). Quantitation of transverse maxillary dimensions using computed tomography: a methodological and reproducibility study. Eur J Orthod.

[19] Chun-Hsi C (2019). Diagnosis of transverse problems. Semin Orthod.

[20] Tai B, Goonewardene MS, Murray K, Koong B, Islam SM (2014). The reliability of using postero-anterior cephalometry and cone-beam CT to determine transverse dimensions in clinical practice. Aust Orthod J.

[21] Abate A, Ugolini A, Maspero C, Silvestrini-Biavati F, Caprioglio A, Lanteri V (2023). Comparison of the skeletal, dentoalveolar, and periodontal changes after Ni-Ti leaf spring expander and rapid maxillary expansion: a three-dimensional CBCT based evaluation. Clin Oral Invest.

[22] Koo Y-J, Choi S-H, Keum B-T, Yu H-S, Hwang C-J, Melsen B (2017). Maxillomandibular arch width differences at estimated centers of resistance: comparison between normal occlusion and skeletal class III malocclusion. Korean J Orthod.

[23] Miner RM, Al Qabandi S, Rigali PH, Will LA (2015). Cone-beam computed tomography transverse analyses. Part 2: measures of performance. Am J Orthod Dentofac Orthop.

[24] Miner RM, Al Qabandi S, Rigali PH, Will LA. Cone-beam computed tomography transverse analysis. Part I: normative data. American Journal of Orthodontics and Dentofacial Orthopedics: Official Publication of the American Association of Orthodontists, its Constituent societies, and the American Board of Orthodontics. 2012;142(3):300–7. 10.1016/j.ajodo.2012.04.014 22920695

[25] Hwang S, Song J, Lee J, Choi YJ, Chung CJ, Kim KH (2018). Three-dimensional evaluation of dentofacial transverse widths in adults with different sagittal facial patterns. Am J Orthod Dentofac Orthop.

[26] Kong L, Liu Y, Zhou X, He H, Liu Z (2024). Responsiveness of three measurements in cone-beam computed tomography transverse analyses during both tooth-supported and mini-screw-assisted rapid maxillary expansion. Angle Orthod.

[27] Zhang C, Guo Q, Liu W, Tang Y, Yuan R. Maxillary transverse deficiency diagnosed by 3 methods and its relationship with molar angulation in patients with skeletal Class III malocclusion. American Journal of Orthodontics and Dentofacial Orthopedics: Official Publication of the American Association of Orthodontists, Its Constituent Societies, and the American Board of Orthodontics. 2023;164(1). 10.1016/j.ajodo.2022.09.015 36813651

[28] Kapetanovic A, Theodorou CI, Berge SJ, Schols J, Xi T (2021). Efficacy of Miniscrew-assisted Rapid Palatal Expansion (MARPE) in late adolescents and adults: a systematic review and meta-analysis. Eur J Orthod.

[29] de Oliveira CB, Ayub P, Ledra IM, Murata WH, Suzuki SS, Ravelli DB (2021). Microimplant assisted rapid palatal expansion vs surgically assisted rapid palatal expansion for maxillary transverse discrepancy treatment. Am J Orthod Dentofac Orthop.

[30] Faul F, Erdfelder E, Buchner A, Lang A-G (2009). Statistical power analyses using G*Power 3.1: tests for correlation and regression analyses. Behav Res Methods.

[31] Zhang CX, Tan XM, Wu W, Liu H, Liu Y, Qu XR (2021). Reliability of 2 methods in maxillary transverse deficiency diagnosis. Am J Orthod Dentofac Orthop.

[32] Ma T, Wang YH, Zhang CX, Liu DX (2023). A novel maxillary transverse deficiency diagnostic method based on ideal teeth position. BMC Oral Health.

[33] Major PW, Johnson DE, Hesse KL, Glover KE (1996). Effect of head orientation on posterior anterior cephalometric landmark identification. Angle Orthod.

[34] Ghafari J, Cater PE, Shofer FS. Effect of film-object distance on posteroanterior cephalometric measurements: suggestions for standardized cephalometric methods. American Journal of Orthodontics and Dentofacial Orthopedics: Official Publication of the American Association of Orthodontists, its Constituent societies, and the American Board of Orthodontics. 1995;108(1):30–7. 10.1016/s0889-5406(95)70063-3 7598102

[35] Legrell PE, Nyquist H, Isberg A (2000). Validity of identification of gonion and antegonion in frontal cephalograms. Angle Orthod.

[36] Leonardi R, Annunziata A, Caltabiano M (2008). Landmark identification error in posteroanterior cephalometric radiography. A systematic review. Angle Orthod.

[37] Major PW, Johnson DE, Hesse KL, Glover KE (1994). Landmark identification error in posterior anterior cephalometrics. Angle Orthod.

[38] Malkoc S, Sari Z, Usumez S, Koyuturk AE (2005). The effect of head rotation on cephalometric radiographs. Eur J Orthod.

[39] Wilcox AJ, Cortese M, McConnaughey DR, Moster D, Basso O (2021). The limits of small-for-gestational-age as a high-risk category. Eur J Epidemiol.

